# Dependence of Electrochemical Properties of MK-40 Heterogeneous Membrane on Number of Adsorbed Layers of Polymers

**DOI:** 10.3390/membranes12020145

**Published:** 2022-01-25

**Authors:** Olesya Rybalkina, Kseniia Tsygurina, Konstantin Sabbatovskiy, Evgeniy Kirichenko, Vladimir Sobolev, Ksenia Kirichenko

**Affiliations:** 1 Physical Chemistry Department, Kuban State University, 149 Stavropolskaya St., 350040 Krasnodar, Russia; olesia93rus@mail.ru (O.R.); kseniya_alx@mail.ru (K.T.); 2 Frumkin Institute of Physical Chemistry and Electrochemistry RAS, 31 Leninsky Prospect, 119071 Moscow, Russia; sabbat07@mail.ru (K.S.); vsobolev@phyche.ac.ru (V.S.); 3 Department of Public and International Law, Kuban State Agrarian University Named after I.T. Trubilin, 13 Kalinina St., 350004 Krasnodar, Russia; lbcs@yandex.ru

**Keywords:** ion exchange membrane, membrane modification, layer by layer coating, voltammetry, limiting current density, electroconvection, monovalent selectivity

## Abstract

The creation of monovalent selective ion exchange membranes benefits the desalination of surface waters by removing interfering monovalent ions while preserving polyvalent ionic nutrients. Studies of a promising method of layer-by-layer adsorption of polymers for the creation of monovalent selective coatings note a significant effect of the number of formed layers and of the nature of the external layer on the properties of the resulting membranes. This article reports the changes in properties of layer-by-layer coated heterogeneous membranes occurring at increasing numbers of layers that are attributed to the supposed intermixing of polymers between the layers, namely dependence of limiting current densities determined from i-V curve, enhanced electroconvection that was attributed to the appearing electrical heterogeneity of the surface, and the decreasing monovalent selectivity in electrodialysis of mixed NaCl + CaCl_2_ solution (from 1.33 to about 1) between the samples with five and six to eight layers of polymers.

## 1. Introduction

Agriculture is the largest consumer of water, accounting for about 70% of global freshwater withdrawals. The pure water shortage leads to the need for desalination and purification of brackish, polluted water or sea water to obtain fresh water suitable for irrigation.

There are studies showing that the desalination of brackish or saline waters and purification of contaminated water for the production of irrigation water is considered economically feasible in the case of intensive production [1] or dry climate [1,2]. Water can be purified or desalted using a number of membrane technologies [3,4,5,6,7]; there are new designs for membranes for metal removal from wastewater [8] and there are membrane technologies that could aid in recuperation of energy for desalination process [9], but the disadvantage is that widely used membrane technologies, including reverse osmosis, nonselectively reduce the total mineralization [10]. This means that ionic nutrients required for the normal functioning of living organisms, such as Ca^2+^, Mg^2+^, SO_4_^2−^ and PO_4_^3−^ are removed together with undesirable monovalent ions such as Na^+^ and Cl^−^, which can lead to nutrient deficiencies [11]. Therefore, remineralization is required if such water is supplied for irrigation [12]. The remineralization is done either through the addition of fertilizers [13], increasing the cost of water treatment, and the presence of monovalent ions in fertilizers can bring the desalted water back to the state when the concentration of monovalent ions is excessive, or through mixing demineralized and source water [1], which can also lead to an excess of the permissible concentration of monovalent ions. Therefore, selective demineralization using selective electrodialysis carried out using monovalent selective membranes was proposed [13] for agricultural producers with high requirements for water quality, such as greenhouses.

The use of electrodialysis for such goals has previously encountered problems of preferential transport of polyvalent ions over monovalent ions by many commercial monopolar membranes. For example, the selectivity of Na^+^ transport over Ca^2+^ through a commercial Neosepta CMX membrane is 0.64 [14]; the Russian heterogeneous MK-40 membrane also preferentially transports polyvalent ions [15]. The first major studies aimed at modification to solve this problem were apparently carried out by the Japanese membrane manufacturer Tokuyama Soda (currently Astom) [16], which was also involved in the development of a process for obtaining table salt from sea water. The approach proposed by this company consisted in the deposition of a polyelectrolyte layer on the ion exchange membrane, and the polar groups of the membrane should have been oppositely charged to the polar groups in the membrane bulk. However, the authors noted that in this way only a modest selectivity can be achieved. Later, cross-linked polymers [17] which form a sieve-like pattern and an increase in the hydrophobicity of the membrane were also employed in an attempt to increase the selectivity that also did not lead to the achievement of the selectivity values required for practice. Other methods of membrane modification to increase the monovalent selectivity are described in a recent review [18].

That review, as well as a number of other works, notes that an easy and flexible way to form a monovalent selectivity is a layer-by-layer assembly of polymers, when a system is formed in which cation exchange layers alternate with anion exchange ones [19]. In this case, the selectivity arises as a result of a stronger Donnan exclusion of polyvalent ions compared with the exclusion of monovalent ions by layers of the polymer that are of the same charge as the transported ions [20]. The advantages of the method are the possibility of choosing an anchoring mechanism from the simplest immersion of samples into solutions of various electrolytes with washing stages between them, which utilizes adsorption due to electrostatic interactions, to complicated procedures that allow precise control of the amount of applied material, such as the covalent linking of each new layer (click chemistry), and the possibility of using not only ion exchange membranes [21] but also various porous materials [22] as substrates, as well as very high values of the achieved selectivities reported by some studies.

The article [19] reported that the layer-by-layer adsorption of 11 layers of polyallylamine and sulfonated polystyrene from their aqueous solutions made it possible to achieve a K^+^/Mg^2+^ selectivity exceeding 1000, while the selectivity of the substrate membrane was 1.8. Further studies confirmed an increase in the monovalent selectivity of membranes when using layer-by-layer adsorption, but the achieved selectivities do not reach the order of thousands belonging approximately to the range 1–50 (Na^+^/Mg^2+^ selectivities were 1.7 and 7.8 in [23], Li^+^/Mg^2+^ selectivity was 5.16 in [24], Cl^−^/SO_4_^2−^ selectivity was 1.8 in [25]). It is suggested that the presence of defects in the formed layers [26] and a decrease in the charge density upon swelling of polyelectrolytes, leading to a loss of the barrier effect [27], might be the causes of the formation of samples with a lower selectivity.

From the point of optimization of costs and the magnitude of the achieved monovalent selectivity, it is important to determine how the selectivity depends on the number of formed layers. It was found that an increase in the number of formed layers makes it possible to increase the selective permeability of the membrane, but with a growing number of layers the role of each new layer on the total selectivity decreases. As an example, in a recent review [28] it was reported that an increase in monovalent selectivity with a higher number of adsorbed layers is observed up to 10 layers. However, with an increase in the number of formed layers, the number of defects in the coating increases, and its layered structure can be disturbed as a result of the diffusion of polymer molecules between the layers [29], as a result of which the conduction paths for polyvalent ions can be formed. Speaking about the characteristics of the membrane other than the monovalent selectivity, when each new layer of polymer is applied, a bipolar boundary is formed between it and the previous layer (one of which, due to alternation, will be cation-exchange, and the other—anion-exchange), the presence of which can significantly increase the electrical resistance of the membrane [30] due to the formation during operation of the membrane of a desalted region inside the membrane, in which there are no carriers of electric current, while the generation of H^+^ and OH^−^ ions also gets significantly enhanced since these ions serve as new carriers of electric current. Consequently, to find the optimum selectivity and conductivity, it is of interest to study the dependence of the transport (electrical conductivity, limiting current) and mass transfer (selectivity during desalting mixed solutions) characteristics of membranes on the number of formed polymer layers.

The earlier article demonstrates the possibility of the creation of selective membranes based on MK-40 heterogeneous membranes and focuses on comparison of the resistance, limiting current density and the intensity of generation of H^+^ and OH^−^ ions of the modified membranes with the substrate membrane [31], which is an example of a heterogeneous sulfocationite membrane manufactured by a local company. Samples MK-40+1—MK-40+5 were produced that contained a homogenizing layer of perfluorosulfonic material and one to four alternating layers of polyallylamine and sodium polystyrene sulfonate, their i-V curves were registered in 0.02 M NaCl solution and the monovalent selectivity was determined in a mixed 0.015 M NaCl + 0.0075 M CaCl_2_ solution. The work demonstrated that the membranes possess Na^+^/Ca^2+^ selectivity and that they have electrical resistance, limiting current density and the intensity of generation of H^+^ and OH^−^ ions comparable to those for the original commercial substrate membrane. The next stage of study, reported in the present article, tested the possibility to further increase the selectivity of samples by applying additional layers of polymers, and included i-V curves recorded in solution with polyvalent cation (0.01 M CaCl_2_) into the consideration and further analyzed the effect of increasing the number of applied layers on the properties of the membranes. The aim was to determine if any properties depend on the nature of the external layer and if this dependence continues through the entire range on the studied number of layers, if there is a difference between the shape of i-V curves registered in solutions with monovalent and polyvalent counterion, if there is a maximum of monovalent selectivity achieved for layer-by-layer coating of this cheap heterogeneous membrane, and if the maximum selectivity is closer to that demonstrated by the average layer-by-layer coated samples (1–50) or to that demonstrated by the best layer-by-layer coated samples (over 1000).

For modification, an MK-40 heterogeneous cation exchange membrane was chosen. It is made by the hot rolling of powdered polyethylene and a powdered sulfonated copolymer of styrene and divinylbenzene between polyamide cloths. The advantages of the membrane are the price (several times lower than the price of Nafion membranes), the mechanical strength and the chemical resistance [32], as well as tolerance to drying and re-swelling, which allows drying the membrane to some extent if required for coating. Its disadvantages follow from the presence of more than 50% [33] of components nonconductive to ions—the membrane has a lower limiting current density and a higher resistance than homogeneous membranes [34].

These disadvantages can be leveled by creating a homogenizing layer on the membrane surface; there is evidence of the success of such a modification using cation exchange perfluorosulfone—the Nafion dispersion and its local analogue LF-4SC [33]. In our work, the creation of such a layer also served the purpose of forming a chemically uniform surface on which subsequent modifiers were then adsorbed.

The anion exchange polymer polyallylamine and the cation exchange polymer sodium polystyrene sulfonate were selected based on their wide use in the process of layer-by-layer coating [22,25] and on the values of electrical conductivity of samples measured during previous experiments [31,35]. Also, these modifiers are optimal from the point of view of safety requirements, environmental friendliness and legality of use in the targeted industries. Sodium polystyrene sulfonate does not need additional checks as a modifier since the MK-40 membrane already contains sulfonated poly(styrene-divinylbenzene). Polyallylamine, in contrast to, for example, polyethyleneimine [36], which is also often used to modify ion-exchange membranes in order to increase the selectivity, is not toxic to aquatic organisms and the GHS P273 code, which states to avoid release to the environment, does not apply to it [37]. Also, unlike polyethyleneimine, polyallylamine does not belong to dangerous goods according to ADR/RID and IATA, which facilitates logistics when purchasing a polymer, reducing the total cost of a modified membrane.

## 2. Materials and Methods

Samples were made as follows. First, a commercial MK-40 membrane (manufactured by Shchekinoazot JSC. (Pervomayskiy (Tula Oblast), Russia)), purchased in a dry form, was cut into samples according to the cell size (5 × 5 cm^2^), then the fragments were placed in a concentrated NaCl, in a concentrated CaCl_2_ or in a 2 M NaCl + 1 M CaCl_2_ mixed solution (corresponding salts were purchased from Vekton Inc., St. Petersburg, Russia) which was periodically diluted with distilled water (produced on site) until the salt concentration became centimolar, then the samples were placed in a 0.02 M NaCl solution or a 0.01 M CaCl_2_ solution (for i-V curves registration) or in a 0.015 M NaCl + 0.0075 M CaCl_2_ mixed solution (for monovalent selectivity study) and kept with periodic replacement of the solution until the electrical conductivity of the equilibrating solution of became constant as follows. The 2 L of each solution was prepared and the conductivities were measured with an Expert 002 conductivity meter equipped with a pen-type conductivity cell (Econics-Expert, Moscow, Russia), then the membrane samples were placed in 150 mL of the solution and left under stirring for 24 h (Monday–Friday) or for 48 h (from Saturday to Monday), then the conductivity of the solution above the membrane was measured and checked against the conductivity of the initial solution, if it differed (increased, since the membranes initially were in more concentrated solution), then the solution above the membrane was discarded and replaced with another portion. The time required for the membrane to become equilibrated depends on its nature, including its thickness and ion exchange capacity, and for MK-40 the entire preparation procedure in this study took a week (the membranes were first placed in concentrated solution on Monday morning, the electrical conductivity of the equilibrating solution did not change between Saturday and the next Monday).

After that, the prepared samples were fixed by their edges to the bottom of the Petri dish using adhesive tape, leaving a working window with a size of at least 2.5 × 2.5 cm^2^ in the center of the membranes, from which droplet moisture was removed. Then, 0.5 mL of a 1% dispersion of the LF-4SC perfluorosulfonic material in isopropyl alcohol (the dispersion is produced by Plastpolymer JSC, St. Petersburg, Russia, purchased as a 7.2% dispersion and then diluted with isopropyl alcohol purchased from Vekton Inc., St. Petersburg, Russia) was distributed over the working window and left in air for 30 min to evaporate the solvent and to harden the layer. Then, 100 mL of a polyallylamine (purchased from Sigma-Aldrich (Saint Louis, MO, USA)) dispersion in water with a concentration of 1 g/L was poured into a Petri dish and left for 30 min for adsorption. After that, the dispersion was discarded, and the membrane was rinsed three times with distilled water (produced on site) to remove the unsorbed polyallylamine. After that, 100 mL of sodium polystyrene sulfonate (purchased from Sigma-Aldrich (Saint Louis, MO, USA)) dispersion in water with a concentration of 1 g/L was poured into a Petri dish and left for 30 min for adsorption, after which the sample was rinsed three times again.

**Figure 1:**
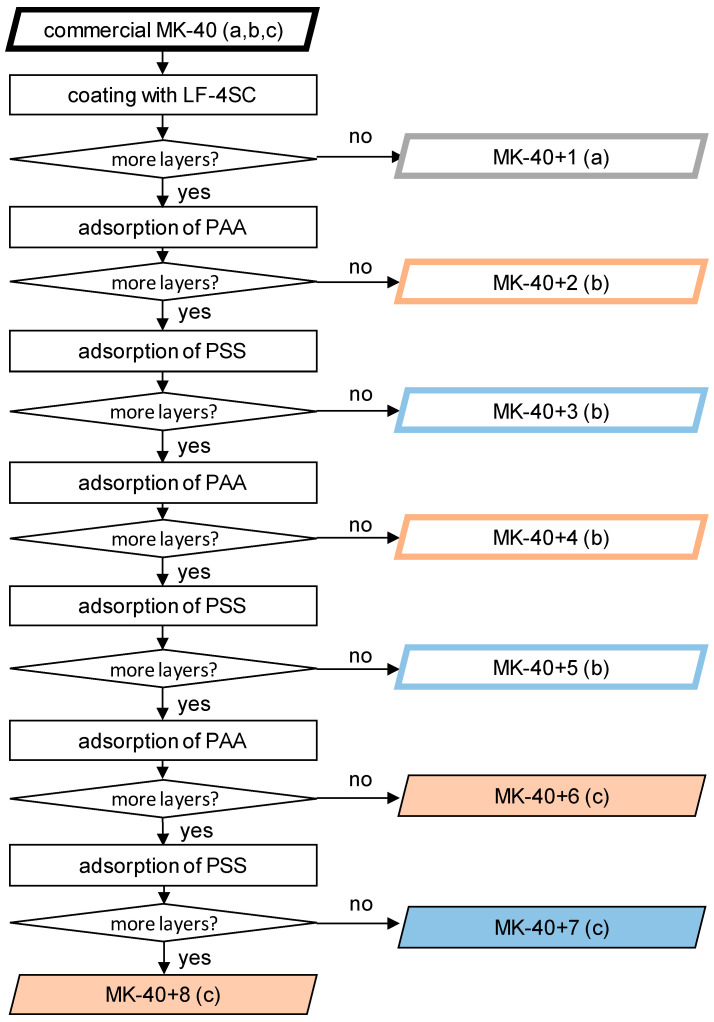
Scheme of modification steps. Filled figures indicate membranes first created for this study, figures with thick frames indicate membranes previously examined. The black frame denotes the MK-40 membrane, the gray one—the MK-40 membrane covered with LF-4SC, the membranes with the upper layer of sodium polystyrene sulfonate (designated as PSS), red—polyallylamine (designated as PAA) are marked in blue. The letters “a”, “b” and “c” represent a series of experiments in which the properties of this membrane were studied.

At this stage, the process was repeated, alternating polymers, until membranes were created consisting of MK-40 (a substrate), a homogenizing layer of LF-4SC, and 5–7 alternating layers of polyallylamine and sodium polystyrene sulfonate. A scheme of the production of samples and the designation of all created membranes are shown in Figure 1. In scheme small letters in brackets denote the series of experiment in which the samples were created and tested. The properties of the membranes were investigated using as a control a sample MK-40 cut from the same sheet as the modified membranes. The MK-40+1 along with its control belong to the series *a*, the data on which were previously published in the article [35]. The MK-40+2, MK-40+3, MK-40+4 and MK-40+5 and corresponding control belong to the series *b*, the thickness, monovalent selectivity and i-V curves of these membranes in a 0.02 M NaCl solution were published in [31], and the i-V curves in a 0.01 M CaCl_2_ solution were not been published previously. The new membranes MK-40+6, MK-40+7 and MK-40+8 along with their control belong to the series *c*, and the properties of these membranes are reported below. For each group of membranes, an MK-40 sample, made from the same sheet that was used as a substrate for modification, was also used as a control; the properties of this sample were studied in the same time period and on the same setups as properties of modified membranes.

The thickness of the coatings on the MK-40+6, MK-40+7 and MK-40+8 membranes was estimated using a JEOL JSM 7500F field emission scanning electron microscope (Jeol, Tokyo, Japan) of the Collective Use Center “Diagnostics of the structure and properties of nanomaterials” of the Kuban State University. Before measurements, the samples were cut to produce cross section, and at mounting in the microscope the samples were vacuumed (and, consequently, the water was removed from them). The thickness of coating was measured at four points for each sample.

**Figure 2:**
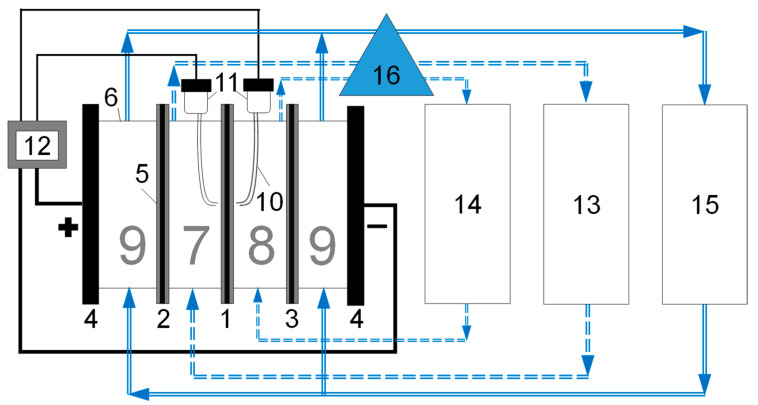
Scheme of the experimental setup in the version used for electrodialysis. 1 is the studied membrane, 2 and 3 are auxiliary anion exchange and cation exchange membranes, respectively, 4 are electrode plates (both cathode and anode were 2 × 2 cm^2^ in area, were made of polished platinum foils (about 0.01–0.02 mm thick) attached to the plexiglass plate by epoxy resin, from the reverse part of the foils copper wires are welded that pass through the plate and are connected to stainless steel plugs from the outer side, the assembly is sealed by the sealant), 5 are rubber inserts (o-rings, 0.08 cm thick in assembled state) that ensure water tightness, 6 are frames made of plexiglass (0.5 cm thick), 7 is a desalination chamber, 8 is a concentration chamber, 9 are electrode chambers, 10 is a Luggin capillary, 11 is Ag/AgCl electrodes, 12 is a power source and a voltmeter, 13 is a circulation tank of a desalination chamber, 14 is a circulation tank of a concentration chamber, 15 is a circulation tank of electrode chambers, 16 is a multichannel pump. A glass electrode for measuring pH and a conductometric cell are located in the circulation tanks, as well as at the outlet from the desalting chamber. When registering the i-V curves, the desalination and concentration chambers are connected to a common circulation tank, and not to separate ones.

Electrodialysis of 100 mL of a mixed solution containing 0.015 M NaCl and 0.0075 M CaCl_2_ was performed in a galvanostatic mode at a current density of 1.5 mA/cm^2^ using a flow-through cell for registration of the i-V curves and mass transport characteristics of ion exchange membranes (Figure 2), and the solution was processed in a circulating mode. All chambers were of the same size and were of a rectangular prism shape. The dimensions of the desalination chamber were 2 cm (length along the desalination path) × 2 cm (width) × 0.66 cm (intermembrane distance), and the diffusion coefficients of NaCl and CaCl_2_ in the solution were taken equal to those at infinite dilution at 25 °C. The solution pumping rate was 0.36 cm/s. For such parameters, the theoretical limiting current density calculated according to Equation (1) [38] is 1.90 mA/cm^2^, therefore, the ratio of the experimental limiting current density to the theoretical one is 0.8, meaning the experiment was made in underlimiting current mode:

(1) $$i_{lim}^{theor}=\frac{F}{\delta }\overset{2}{\underset{k=1}{\sum }}\left(1-\frac{z_{a}}{z_{k}}\right)D_{k}z_{k}C_{k}$$

here *F* is the Faraday constant (9.6485 × 10^6^ mA × s/mol), *δ* is the diffusion layer thickness (in this case equal to 0.025 cm, the details of the calculation are described in [39]), *D_k_*, z*_k_* and *C_k_* are the diffusion coefficient (1.334 × 10^−5^ cm^2^/s for Na^+^ and 0.792 × 10^−5^ cm^2^/s for Ca^2+^), charge and molar concentration of counterion *k*, respectively (*k* = 1,2, the product of *z_k_C_k_* is always 2 × 10^−5^ mol/cm^3^); z*_A_* is the coion charge number (dimensionless).

The electrodialysis desalination continued for 5 h, and each hour an aliquot of 1 mL was taken to determine the concentration of Na^+^ and Ca^2+^ in solution using Shimadzu LC-20 Prominence high-performance liquid chromatograph with a conductometric detector by the Collective Use Center “Ecological and analytical center” of the Kuban State University.

The i-V curves of the studied membranes were recorded in a 0.02 M NaCl solution and in a 0.01 M CaCl_2_ solution (corresponding to the same equivalent concentration of 0.02 mol/L) using the same cell (except that during the registration of i-V curves the desalination and concentration chambers are connected to a common circulation tank, and not to separate ones) in the galvanodynamic mode in the range of current densities 0–5 mA/cm^2^ with a sweep rate of 2.5 μA/(cm^2^/s). The theoretical limiting current density calculated by the Lévêque equation [40] (Equation (2)) was 1.93 mA/cm^2^ in 0.02 M NaCl and 1.84 mA/cm^2^ in 0.01 M CaCl_2_.

(2) ilimtheor=zkCkFDh(T1−t1)[1.47(h2V0LD)13−0.2]

here *z_k_C_k_* is 2 × 10^−5^ mol/cm^3^ for both salts, *F* is the Faraday constant (9.6485 × 10^6^ mA × s/mol), *D* is the salt diffusion coefficient in the solution (1.61 × 10^−5^ cm^2^/s for NaCl and 1.34 × 10^−5^ cm^2^/s for CaCl_2_, respectively); *h* is the intermembrane distance (0.66 cm); *T*_1_ and *t*_1_ are the counterion transport numbers in the membrane and in solution, respectively (*T*_1_ is taken as 1, assuming the membrane to be absolutely selective, *t*_1_ was 0.603 for Na^+^ in NaCl and *t*_1_ was 0.438 for Ca^2+^ in CaCl_2_); *V*_0_ is the linear solution pumping rate (0.36 cm/s); *L* is the length of the desalination path (2 cm).

An equation describing the theoretical current limit was derived to describe the electrodiffusion of salt to a homogeneous membrane, and this description does not consider the presence of multiple layers within the membrane. In the present work this equation is used rather as a means for normalizing the results of experiments carried out under different conditions. An equation describing the limiting current of a membrane with a larger number of layers was deduced in [41], and i-V curve of a composite bilayer membrane was modeled in [42].

The i-V curves were analyzed as follows:

To assess the resistance of the membrane, the i-V curves were also recorded in the configuration of the setup in which the studied membrane was removed (while all the seals normally holding the membrane and providing water tightness that are indicated in Figure 2 by the number 5 remained in their positions). From the i-V curves recorded with and without a membrane, the slope of the initial part of the ohmic region was determined to calculate the resistance of the solution and the resistance of the solution with the membrane, then the resistance of the membrane was calculated from their difference (Equation (3)).

(3) $$R_{m}=R_{m+s}-R_{s}=\frac{1}{\mathrm{initial}\;\mathrm{slope}\;\mathrm{of}\;iV_{m+s}}-\frac{1}{\mathrm{initial}\;\mathrm{slope}\;\mathrm{of}\;iV_{s}}$$

here the initial slope is calculated for curves built in amperes vs. volts coordinates

The experimental limiting current density was determined by plotting tangents to the so-called ohmic section and to the section plateau of the i-V curve and then finding the point of their intersection.

To be able to compare the data recorded using cells of different configurations or for solutions of different compositions, i-V curves were also plotted in other coordinates. The abscissa plotted the reduced potential drop calculated from the experimentally recorded potential drop between the Luggin capillaries after subtracting from it the product of the current and the ohmic resistance of the solution with the membrane (Equation (4)):

(4) $$\Delta \varphi^{\prime }=\Delta \varphi -IR_{m+s}$$

here all potential drops are expressed in volts, *I* is the current in amperes, *R_m_*_+*s*_ is the resistance expressed in ohms.

The ordinate plotted the dimensionless current density equal to the ratio of experimental current density to the theoretical limiting current density calculated by the Lévêque equation.

## 3. Results

### 3.1. Thickness

The membrane thicknesses determined by scanning electron microscopy are given in Table 1.

### 3.2. i-V Curves

**Figure 3:**
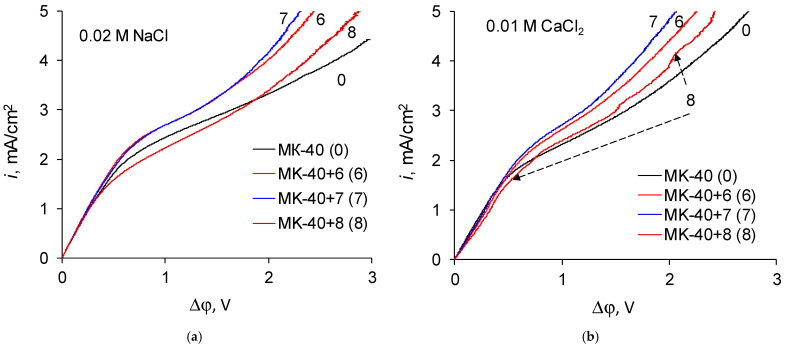
The i-V curves of the membranes in the coordinates “current density vs. potential drop between the Luggin capillaries” in (**a**) 0.02 M NaCl solution; (**b**) 0.01 M CaCl_2_ solution.

The i-V curves of the membranes are given in the coordinates “current density vs. potential drop between the Luggin capillaries” in Figure 3.

**Figure 4:**
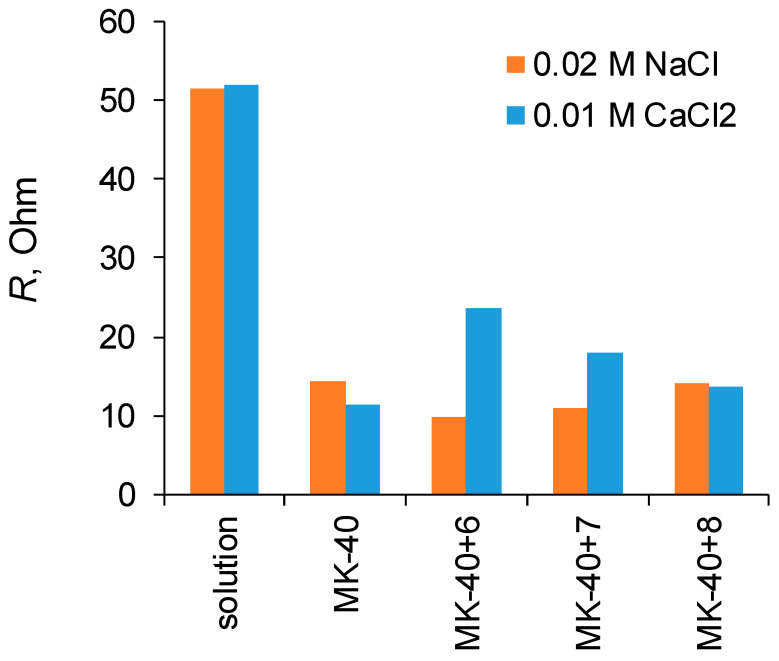
Resistances of membranes *R_m_* and solutions *R_s_* determined from the i-V curves of the membranes in solution and the i-V curves of the solution enclosed between Luggin capillaries in the absence of a membrane between them.

A comparison of the resistances of the layer solution enclosed between the Luggin capillaries determined from the slopes of the initial sections of the i-V curves and the resistance of membranes calculated from the difference in resistance of a system containing a membrane and a system without a membrane are shown in Figure 4. It can be seen that in all cases the application of polymers did not lead to such an increase in the membrane resistance that it would become comparable to the resistance of the layer of solution.

### 3.3. Monovalent Selectivity

**Figure 5:**
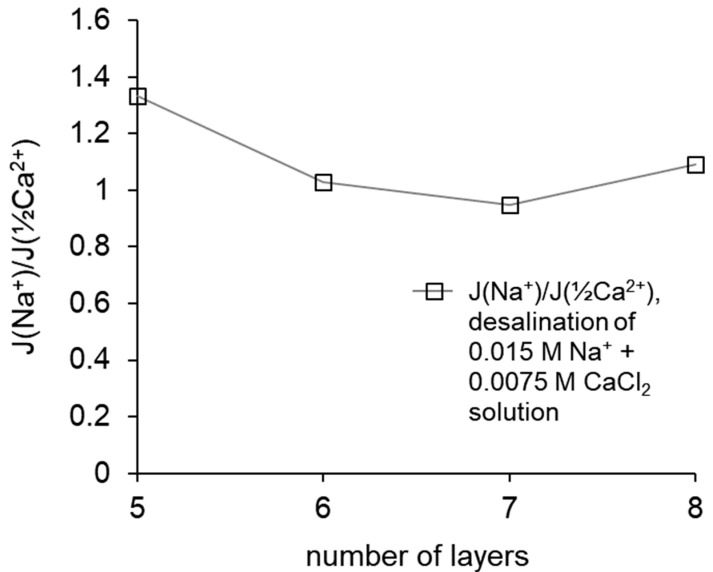
The ratio of the fluxes of Na^+^ and the equivalent (½) of Ca^2+^ through the studied membranes during electrodialysis, calculated as the ratios of the slope of the dependence of the concentration of these ions in the desalination channel on time, vs. the number of formed layers of polymers.

To determine the monovalent selectivity of ion transport from the data of chromatographic determination, changes in the concentration of ions in the course of electrodialysis desalination were determined and their ratios were calculated, which is also the ratio of the fluxes of these ions. Comparison of the ratio of ion fluxes with the ratio calculated for the membrane that demonstrated the best selectivity among the samples produced at the previous stage of the study is made in Figure 5. The previously created membrane with five formed layers of polymers demonstrated greater selectivity; the selectivity decreased upon raising the number of layers to six and then weakly depended on the number of created layers.

## 4. Discussion

### 4.1. Thickness

The thicknesses of the coatings determined using scanning electron microscopy of dry membranes are significantly lower than those previously determined using a micrometer for swollen membranes with a smaller number of formed layers formed by the same technique (the maximum estimate of the thickness of a dry coating consisting of 8 polymer layers on MK-40+8 is 5 μm, while the minimum estimate of the thickness of 5 swollen polymer layers on the corresponding membrane is 10 μm), which can be explained by the high moisture content of the applied layers. The difference in thickness between membranes MK-40+6 and MK-40+8 confirms the success of the formation of new layers of polymers. These layers seem thicker than the ones reported for layer-by-layer systems created at homogeneous membranes. For example, it follows from [19] that the thickness of layers at the Nafion membrane were in the magnitude of tens of nanometers.

### 4.2. i-V Curves

**Figure 6:**
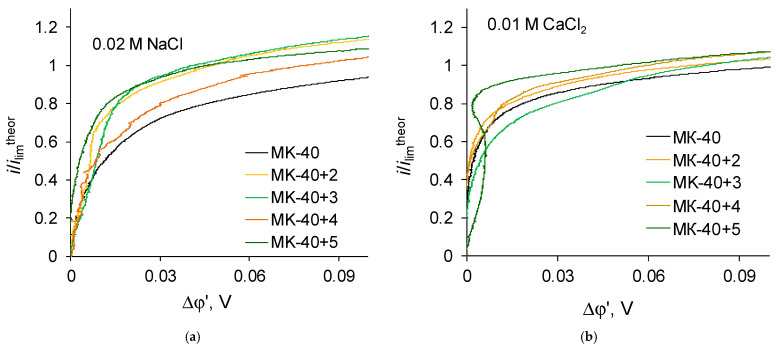
Magnified initial sections of i-V curves registered in (**a**) 0.02 M NaCl solution; (**b**) 0.01 M CaCl_2_ solution for membranes with lesser number of formed layers of polymers plotted in the “current density vs. the potential drop between the Luggin capillaries” coordinates.

The shape of i-V recorded in a 0.02 M NaCl solution for a commercial MK-40 membrane and for modified membranes is typical for monopolar membranes. In the curves, an initial, approximately linear, section, a section of an inclined plateau and a section of overlimiting currents can be distinguished; it is possible to determine the singular limiting current. The curves recorded in a 0.01 M CaCl_2_ solution are also similar to typical curves when plotted in the “current density vs. the potential drop between the Luggin capillaries” coordinates. However, if the curves are built in the “dimensionless current density vs. the reduced potential drop” coordinates, an additional feature can be distinguished. Let us first present the enlarged sections of the underlimiting currents from the i-V curves of the MK-40+2—MK-40+5 membranes (Figure 6):

The shown sections of the curves are similar to each other, with one exception—on the curve recorded in a CaCl_2_ solution for the MK-40+5 membrane, there is a section of negative differential resistance (the curve turns to the left), while the maximum decrease in the potential drop in this section is about 0.01 V.

**Figure 7:**
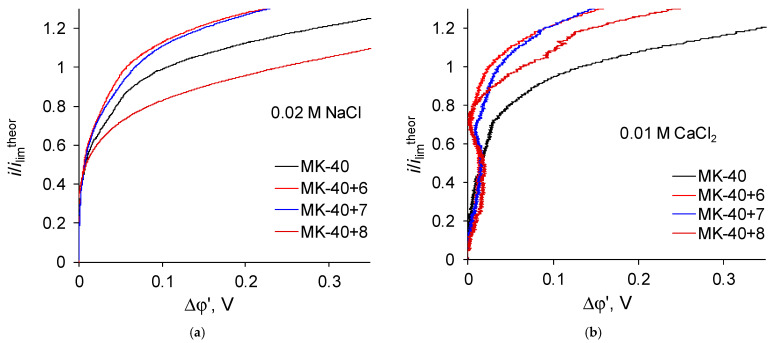
Magnified initial sections of i-V curves registered in (**a**) 0.02 M NaCl solution; (**b**) 0.01 M CaCl_2_ solution for membranes with greater number of formed layers of polymers plotted in the “current density vs. the potential drop between the Luggin capillaries” coordinates.

Let us now regard the curves recorded for membranes with a larger number of formed layers (Figure 7):

Zones of negative differential resistance are also present in the i-V curves of these membranes recorded in a CaCl_2_ solution, while the decrease in potential drop grows and reaches to about 0.02 V. Note also that the deviation is practically independent of the nature of the outer layer, as the curves recorded for the MK-40+6 and MK-40+7 membranes are very close.

Such zones in i-V curves were shown earlier in [43], where their appearance was associated with the impact of equilibrium electroconvection, which is a vortex motion of a solution near the membrane surface in underlimiting current modes as a result of the action of an external electric field on a quasi-equilibrium electric double layer. It is indicated in [44] that the development of equilibrium electroconvection can be caused by local inhomogeneities in the properties of the membrane. In [45], the electroconvection in a microfluidic channel is discussed, the sign of charge of the walls of which at some point inverts to the opposite, and it is concluded that electroconvective vortices are formed in the solution near the point at which the sign of the surface charge changes.

The fact that the deviations of the curve are present only in the CaCl_2_ solution can be explained both by the known formation of larger convective vortices in the CaCl_2_ solution as compared to NaCl solution, which is explained by the large hydrated radius of the counterion and is shown, for example, in [46], and by the assumed stronger repulsion of Ca^2+^ ions from the positively charged layers in the coating.

More interesting is the fact that the described zones appear on the i-V curves of membranes starting only from MK-40+5, and their depth increases upon going to samples with a large number of adsorbed layers of polymers. This indicates a change in some property of the membrane with an increase in the number of formed layers, which is not related to the nature of the outer layer. Taking into account the dependence of the development of electroconvection on the presence of electric heterogeneity of the surface and evidences that the layered structure of layer-by-layer coated membranes can be disturbed when the number of layers increases, it can be hypothesized that the appearance of characteristic areas is associated with the formation of areas with different charges on the membrane surface as a result of the formation of defects and mixing of layers.

Equilibrium electroconvection causes stirring of the solution in the depleted diffusion boundary layer and ensures delivery of a more concentrated solution to the membrane surface. Hence the development of electroconvection should lead to an increase in the experimental limiting current density. Let us consider the dependence of the limiting current densities on the number of formed layers. To exclude the effect of variance between the substrates in different experimental series, the current density of the membranes is given (Table 2) divided by to the experimental limiting current density of the MK-40 membrane, taken as a control, which was determined separately for each series of created membranes.

It can be seen that at a lesser number of formed layers, the limiting current density depends on the composition of the outer polymer layer. The increase in the limiting current density during the deposition of the first polymer layer is explained by the homogenization of the membrane surface, optimization of the distribution of the electric field lines, a decrease in the “funnel effect” [47], and reduction of concentration polarization. For samples with 1–4 formed polymer layers, it is characteristic that the samples have a higher limiting current density when the outer layer of polymer contains polar groups with the same charge as the polar groups of the substrate membrane (namely, samples with 1 and 3 formed layers of polymer) and samples have a smaller limiting current density when the outer layer of the polymer contains polar groups oppositely charged to the polar groups of the substrate membrane (namely, samples with 2 and 4 layers of polymer). In this range the formation of the system of polymers increases the limiting current density of the modified membranes in comparison with the limiting current density of the substrate membrane, while the increase is more significant for the NaCl solution in comparison with CaCl_2_. The smaller limiting current density through the samples the outer layer of which is oppositely charged to the substrate can be explained from the point of view of the influence of equilibrium electroconvection as well, since the deposition of a layer, the polar groups of which are oppositely charged to the polar groups of the membrane volume, reduces the absolute value of the charge of the quasi-equilibrium electric double layer [43].

For a larger number of formed layers, the limiting current density ceases to depend on the nature of the outer layer. In this case, in a NaCl solution, the limiting current density of these membranes is approximately equal to the limiting current density through a membrane with five formed layers, and in a CaCl_2_ solution the limiting current density through membranes with six and seven formed layers noticeably exceeds the limiting current density through a membrane with five formed layers. Note that an increase (or a weaker decrease) in the limiting current density is in good agreement with the presence of sections of negative differential resistance in the sections of underlimiting currents of the i-V curves of the corresponding membranes and with the value of the decrease of the potential drop. Considering that there is information that equilibrium electroconvection can increase the experimental limiting current density, the values of the limiting current density can be considered as another indirect evidence of a more intensive electroconvection in systems with MK-40+5, MK-40+6 and MK-40+7 membranes desalting a 0.01 M CaCl_2_ solution.

The i-V curves of the MK-40+8 membrane show signs characteristic of the intensive development of both equilibrium (the presence of a region of negative differential resistance, Figure 6) and nonequilibrium electroconvection (oscillations of a potential drop in overlimiting current modes [48]), especially noticeable in the case of a CaCl_2_ solution. These features are usually combined with an increased mass transport of salt ions; however, the limiting density of salt counterion through this membrane is much lower than the limiting current density through the MK-40+7 membrane and other studied membranes, including the MK-40 heterogeneous commercial membrane. Such a decrease would hinder the working efficiency of the MK-40+8 membrane if it were mounted in a real electrodialyzer, since the operating current of the apparates working in galvanostatic mode is chosen from underlimiting currents, and the MK-40+8 membrane would allow lower performance than MK-40+6, MK-40+7 or even the commercial MK-40 membrane. Considering the possible causes of the decrease of the limiting current density, the resistances determined from the i-V curves of the MK-40+7 and MK-40+8 membranes are quite close, and although the i-V curve of the MK-40+8 membrane visually has a lower slope than the i-V curves of other modified membranes, it is generally higher than the slope of the i-V curve of MK-40. Thus, there is no obvious reason for the decrease in the limiting current of the membrane with the maximum number of formed polymer layers. It can be assumed that the value of the limiting current in this case could be influenced by the increased thickness of the coating (in [49], the dependences of the transport numbers of salt counterions through a bilayer asymmetric bipolar membrane on thickness of layer polar groups of which are charged oppositely to the polar groups of the substrate is shown and even at thickness of 10 μm the transport number of salt counterions decreased to 0.14); however, in this case, the reason for the sharp change in properties upon going from MK-40+7 to MK-40+8 is unclear. It can also be assumed that in this case the factor that the outer layer of the polymer is charged opposite to membrane bulk, and the factor that a sufficient number of bipolar boundaries have formed in the polymer structure to block the transport of salt counterions, or a combination of these two factors with an increase in the thickness of the coating becomes important, however, at the moment there is not enough data to make a sure conclusion about such mechanisms.

**Figure 8:**
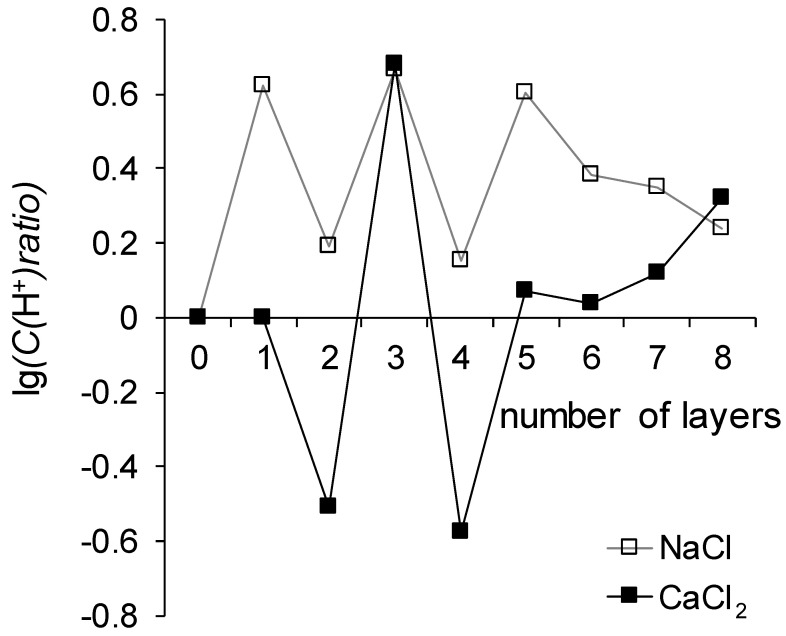
Dependence of the difference between the maximum pH difference recorded during the registration of the i-V curves of the control membranes and the maximum pH difference recorded during the registration of the i-V curves of the tested membranes.

Since the substrates used in different series of experiments were different, possibly affecting the comparison, to discuss the generation of H^+^ and OH^−^ ions, let us present the experimental data on the maximum achieved pH difference between the outlet and inlet of the desalination chamber during the recording of i-V curves in the following form: from the maximum value recorded for channels with a control sample the maximum value recorded for channels containing the membrane under study was subtracted. Taking into account the definition of pH, the calculated values show the logarithm of the ratio of the concentration of H^+^ ions in the solution passing through the chamber with the modified membrane to the concentration of H^+^ ions in the solution passing through the chamber with the corresponding control membrane:

(5) $$\log_{10}C_{H^{+}}\mathrm{ratio}=\Delta {\mathrm{pH}}_{\mathrm{MK}\text{-}40}-\Delta {\mathrm{pH}}_{x}\approx -\left(\log_{10}{\left(C_{H^{+}}\right)}_{\mathrm{MK}\text{-}40}-\log_{10}{\left(C_{H^{+}}\right)}_{x}\right)=\log_{10}\frac{{\left(C_{H^{+}}\right)}_{x}}{{\left(C_{H^{+}}\right)}_{\mathrm{MK}\text{-}40}}$$

here ΔpH is the pH difference between the outlet and inlet of the desalination chamber, the subscript MK-40 means the desalination chamber contains the MK-40 membrane, the subscript *x* means the desalination chamber contains the membrane containing as many layers of polymers (counting the LF-4SC) as indicated on the abscissa of Figure 8.

Note that the desalination chamber is formed by two membranes, one anion exchange and one cation exchange, and the generation of H^+^ and OH^−^ ions occurs on both membranes. The reaction proceeding on the cation exchange membrane delivers OH^−^ ions to the desalination chamber, the reaction proceeding on the anion exchange membrane delivers H^+^ ions to the desalting channel, and the final pH difference is determined by the rates of these reactions. Since the studied membranes are cation exchange, the acceleration of the reaction of generation of H^+^ and OH^−^ ions on these membranes will supply OH^−^ ions to the desalination chamber, as a result of which the concentration of H^+^ ions in it will decrease, and, consequently, the value of log_10_C(H^+^)ratio will decrease as well; suppression of this reaction will lead to an increase in the log_10_C(H^+^)ratio.

It follows from Figure 8 that the generation of H^+^ and OH^−^ ions proceeds more intensively in the system that treats the CaCl_2_ solution as compared to the NaCl solution. This can be explained by the ability of Ca^2+^ ions to participate in the protolysis reaction with the formation of Ca(OH)^+^ and H^+^, which leads to an acceleration of the reaction of generation of H^+^ and OH^−^ ions.

A pattern can also distinguish that the reaction of generation of H^+^ and OH^−^ sharply accelerates in systems with MK-40+2 and MK-40+4 membranes in comparison with neighboring membranes. The outer layer of the polymer on both membranes is polyallylamine, a primary amine, the functional groups of which, according to a series of catalytic activity in the reaction of generation of H^+^ and OH^−^ ions [50], hasten this reaction a hundred times more actively than sulfonic groups of sodium polystyrene sulfonate, which is the outer layer of membranes MK-40+3 and MK-40+5. Note that such a sharp increase does not occur in the case of MK-40+6 and MK-40+8 membranes. The values of the maximum pH shift of the MK-40+6, MK-40+7 and MK-40+8 membranes can be described as lying in between the values determined for MK-40+2, MK-40+4 and for MK-40+3, MK-40+5. This observation is one more piece of evidence that when the number of formed layers does not exceed five, a certain property of the membrane depends on the composition of the outer layer of the polymer, and with an increase in the number of formed layers, the significance of the dependence of the property on the composition of the outer layer of the polymer decreases.

### 4.3. Monovalent Selectivity

The calculated ratios of the fluxes of Na^+^ and (½) Ca^2+^ equivalents through the modified membranes show that the highest selectivity value was achieved by the MK-40+5 membrane, then it decreased upon passing to MK-40+6, then stayed almost constant with possible slight increase for MK-40+8. A decrease in selectivity can also be a consequence of a change in the structure of the coating; in particular, the result of mixing of layers and the formation of defects, which is consistent with the assumption of an increased electrical inhomogeneity of membranes, which also explains the enhanced electroconvection and disappearing dependence of pH on composition of the outer layer.

Note that despite the slight increase in selectivity for MK-40+8 in comparison with MK-40+7, the selectivity of the latter still does not reach the values calculated for MK-40+5, and the decrease in the limiting current density found for MK-40+8 makes it impractical to further search for a membrane with a higher selectivity through the increase in the number of adsorbed layers.

The determined Na^+^/Ca^2+^ selectivities lay in range 1.0–1.35, which agrees with some findings in other articles (Na^+^/Mg^2+^ selectivities were 1.7 in [23], Cl^−^/SO_4_^2−^ selectivity was 1.8 in [25]), but certainly leaves room for the improvement of monovalent selectivity as compared with [19], where K^+^/Mg^2+^ selectivity exceeded 1000. In [19] a (relatively) smooth homogeneous Nafion membrane was used as a substrate; it might be that layers with smaller number of defects are formed at homogeneous membranes as compared with heterogeneous ones, but the cheapness of heterogeneous membranes in comparison with the Nafion membrane makes the search for improvement of monovalent selectivity achieved by layer-by-layer adsorption of polymers at heterogeneous membranes a promising direction of further studies.

## 5. Conclusions

Modified membranes based on the MK-40 heterogeneous membrane were created, containing a homogenizing layer of perfluorosulfone material LF-4SC and adsorbed layers of polyallylamine and sodium polystyrene sulfonate. It is shown that the electrical resistance of the modified membranes is comparable to the resistance of the substrate membrane, and the limiting current densities of salt ions through these membranes exceed the limiting current density through the MK-40 membrane in the case when the number of formed layers does not exceed eight.

A number of properties of modified membranes, such as the value of the limiting current and the intensity of generation of H^+^ and OH^−^ ions, depend on the composition of the outer layer when a number of formed layers is small, but this dependence disappears when the number of formed layers reaches five. Intense equilibrium electroconvection in systems with modified membranes treating a 0.02 M CaCl_2_ solution, on the contrary, first appears when the number of formed layers reaches five. The maximum monovalent selectivity was shown for a membrane with five formed layers of polymer. It can be hypothesized that with a small number of formed layers, their structure is retained, making it possible to observe the dependence of the properties of the samples on the composition of the outer layer, and when a larger number of layers are applied, the layer-by-layer arrangement of polymers is disturbed and coating defects are formed, creating areas with different signs of the charge of the polar groups, which intensifies electroconvection and unifies the rate of generation of H^+^ and OH^−^ ions between membranes, and creating the conduction paths for polycharged ions in the bulk of the coating. These findings might serve as additional evidence to existing claims that the application of the first layer is the most important in formation of monovalent selectivity and that the adsorption of the following layers gives decreasing gains. It also would mean that the improvement of selectivity achieved through layer-by-layer adsorption should be done not in extensive way through increasing the number of layers, but in an intensive way through the formation of defect-free coating, possibly by decreasing the thickness of each layer by reduction of the adsorbed material or by a search for a materials structure which would allow for such assembly, for example, the materials with higher charge.

The possible direction for future studies is confirmation of changes in the structure of layers.

## Figures and Tables

**Table 1:** Entire thickness of the coating (LF-4SC and the layers adsorbed on it) on MK-40+6, MK-40+7 and MK-40+8 membranes.

Membrane	Thickness of Coating, μm
MK-40+6	3.24 ± 0.42
MK-40+7	3.74 ± 0.53
MK-40+8	4.41 ± 0.51

**Table 2:** Experimental limiting of current densities of the MK-40 membrane and modified samples, and the ratios of these current densities to the limiting current density of a MK-40 membrane used as a substrate in corresponding series.

Membrane	Series	Top Layer	LCD * in 0.02 M NaCl, mA/cm^2^	LCD Ratio, 0.02 M NaCl	LCD * in 0.01 M CaCl_2_, mA/cm^2^	LCD Ratio, 0.01 M CaCl_2_
MK-40	a	MK-40 (has SO_3_^−^)	1.81	1	1.70	1
	b		1.85	1	1.76	1
	c		2.06	1	1.78	1
MK-40+1	a	LF-4SC	2.16	1.19	1.87	1.10
MK-40+2	b	PAA	2.1	1.14	1.84	1.05
MK-40+3	b	PSS	2.2	1.19	1.87	1.06
MK-40+4	b	PAA	2.12	1.15	1.90	1.08
MK-40+5	b	PSS	2.02	1.09	1.85	1.05
MK-40+6	c	PAA	2.28	1.11	2.19	1.23
MK-40+7	c	PSS	2.28	1.11	2.20	1.24
MK-40+8	c	PAA	1.60	0.78	1.40	0.79

* LCD stands here for experimental limiting current density.

## Data Availability

The data presented in this study are available on request from the corresponding author.
